# Polycomb repressive complex 2 in adult hair follicle stem cells is dispensable for hair regeneration

**DOI:** 10.1371/journal.pgen.1009948

**Published:** 2021-12-14

**Authors:** Pooja Flora, Meng-Yen Li, Phillip M. Galbo, Maider Astorkia, Deyou Zheng, Elena Ezhkova

**Affiliations:** 1 Black Family Stem Cell Institute, Department of Cell, Developmental, and Regenerative Biology, Icahn School of Medicine at Mount Sinai, New York, New York, United States of America; 2 Department of Genetics, Albert Einstein College of Medicine, New York, New York, United States of America; 3 Departments of Genetics, Neurology, and Neuroscience, Albert Einstein College of Medicine, New York, New York, United States of America; Boston University School of Medicine, UNITED STATES

## Abstract

Hair follicle stem cells (HFSCs) are multipotent cells that cycle through quiescence and activation to continuously fuel the production of hair follicles. Prior genome mapping studies had shown that tri-methylation of histone H3 at lysine 27 (H3K27me3), the chromatin mark mediated by Polycomb Repressive Complex 2 (PRC2), is dynamic between quiescent and activated HFSCs, suggesting that transcriptional changes associated with H3K27me3 might be critical for proper HFSC function. However, functional *in vivo* studies elucidating the role of PRC2 in adult HFSCs are lacking. In this study, by using *in vivo* loss-of-function studies we show that, surprisingly, PRC2 plays a non-instructive role in adult HFSCs and loss of PRC2 in HFSCs does not lead to loss of HFSC quiescence or changes in cell identity. Interestingly, RNA-seq and immunofluorescence analyses of PRC2-null quiescent HFSCs revealed upregulation of genes associated with activated state of HFSCs. Altogether, our findings show that transcriptional program under PRC2 regulation is dispensable for maintaining HFSC quiescence and hair regeneration.

## Author summary

Hair follicle stem cells (HFSCs) have the incredible capacity to cycle through quiescence and activation to fuel the production of hair follicles throughout the life of an organism. During the onset of hair growth, HFSCs undergo transient activation to proliferate but quickly revert to their quiescent state for the remaining of the hair cycle phases. This balance between HFSC activation and quiescence is maintained by establishing a specific transcriptional landscape; however, very little is known about how chromatin-modifying factors, critical regulators of gene transcription, control adult HFSC function. Prior genome mapping studies had shown that H3K27me3, the chromatin mark mediated by Polycomb Repressive Complex 2 (PRC2), is dynamic between quiescent and activated HFSCs, suggesting that transcriptional changes associated with H3K27me3 might be critical for proper HFSC function. Despite this correlation, functional *in vivo* studies elucidating the role of PRC2 in adult HFSCs were lacking. In this study, we show that ablation of PRC2 in the adult HFSCs leads to modest transcriptional changes that are insufficient to alter HFSC fate or function to have physiologically relevant effects, indicating that other chromatin regulatory mechanisms could be safeguarding adult HFSC function and identity.

## Introduction

Hair follicles (HFs) undergo cycles of growth (anagen), destruction (catagen), and rest (telogen) throughout an organism’s lifetime [1]. This cyclical regeneration is fueled by hair follicle stem cells (HFSCs) that reside in the niche called the bulge, where they remain in a quiescent state influenced by the incoming extrinsic inhibitory signals [1,2]. During telogen-to-anagen transition, the dermal papilla (DP), located below the bulge, provides activating cues that signal the HFSCs to briefly proliferate and produce differentiating transit amplifying cells (HF-TAC) that give rise to a new HF before returning to the quiescent state [3]. The balance between stem cell activation and quiescence is maintained by establishing a specific transcriptional landscape [4]. However, very little is known about how chromatin modifying factors, critical regulator of gene transcription, control HFSC function and hair regeneration in the adult skin.

The Polycomb group of proteins are major epigenetic regulators of stem cell fate maintenance and differentiation [5]. Polycomb proteins form two distinct multi-protein complexes, Polycomb Repressive Complex 1 (PRC1) and PRC2, that mediate transcriptional repression via chromatin compaction [6,7]. Specifically, PRC2 complex facilitates the tri-methylation of histone H3 at lysine 27 (H3K27me3) modification, which results in gene repression [8,9].

Loss of PRC2 in the developing epidermis has various phenotypic consequences on epidermal barrier formation and HF morphogenesis [10–12]. However, its physiological function in the adult skin remains unexplored. Prior work has shown that levels of chromatin marks, H3K27me3, H3K4me3 and H3K9me3, are reduced in HFSCs prior to activation, and pharmacological inhibition of de-methylases in the epidermis, including the HFSCs, results in elevated levels of global tri-methylation leading to defective hair regeneration [13]. Notably, genome wide mapping of H3K27me3 in quiescent and activated HFSCs as well as in HF-TACs revealed that while H3K27me3 demarcates key HF-TAC genes in the HFSCs, this histone mark is lost from these genes in the HF-TACs where these genes are active [14]. These correlative studies suggest that PRC2-mediated H3K27me3 represses differentiation committed genes in the HFSCs to not only maintain HFSC quiescence but also to preserve its cell fate identity. While these studies imply that PRC2 and H3K27me3 may govern adult HFSC function, *in vivo* functional studies exploring the role of PRC2 in adult HFSCs are lacking.

In this study, we utilized genetic approaches and genome-wide transcriptional studies to elucidate the role of PRC2 in adult HFSCs. We found that ablation of PRC2 in quiescent HFSCs (qHFSCs) does not alter HFSC quiescence or cause fate switch as speculated from H3K27me3 chromatin mapping studies. Interestingly, RNA-seq studies revealed that loss of PRC2 in qHFSCs resulted in expression of subset of genes that are normally expressed in activated HFSCs (aHFSCs), yet these transcriptional changes are not sufficient to induce phenotypic changes. Lastly, ablation of PRC2 in the HFSCs does not alter homeostatic hair cycle nor does it impact regenerative capacity as PRC2-null HF bulge cells are capable of regenerating new HFs. Taken together, our studies reveal that PRC2 and H3K27me3 play a non-instructive role in the adult HFSCs and there may be redundant or alternate mechanisms in place to preserve HFSC function and fate maintenance.

## Results

### HFSC fate and identity is maintained upon loss of H3K27me3

**Fig 1:**
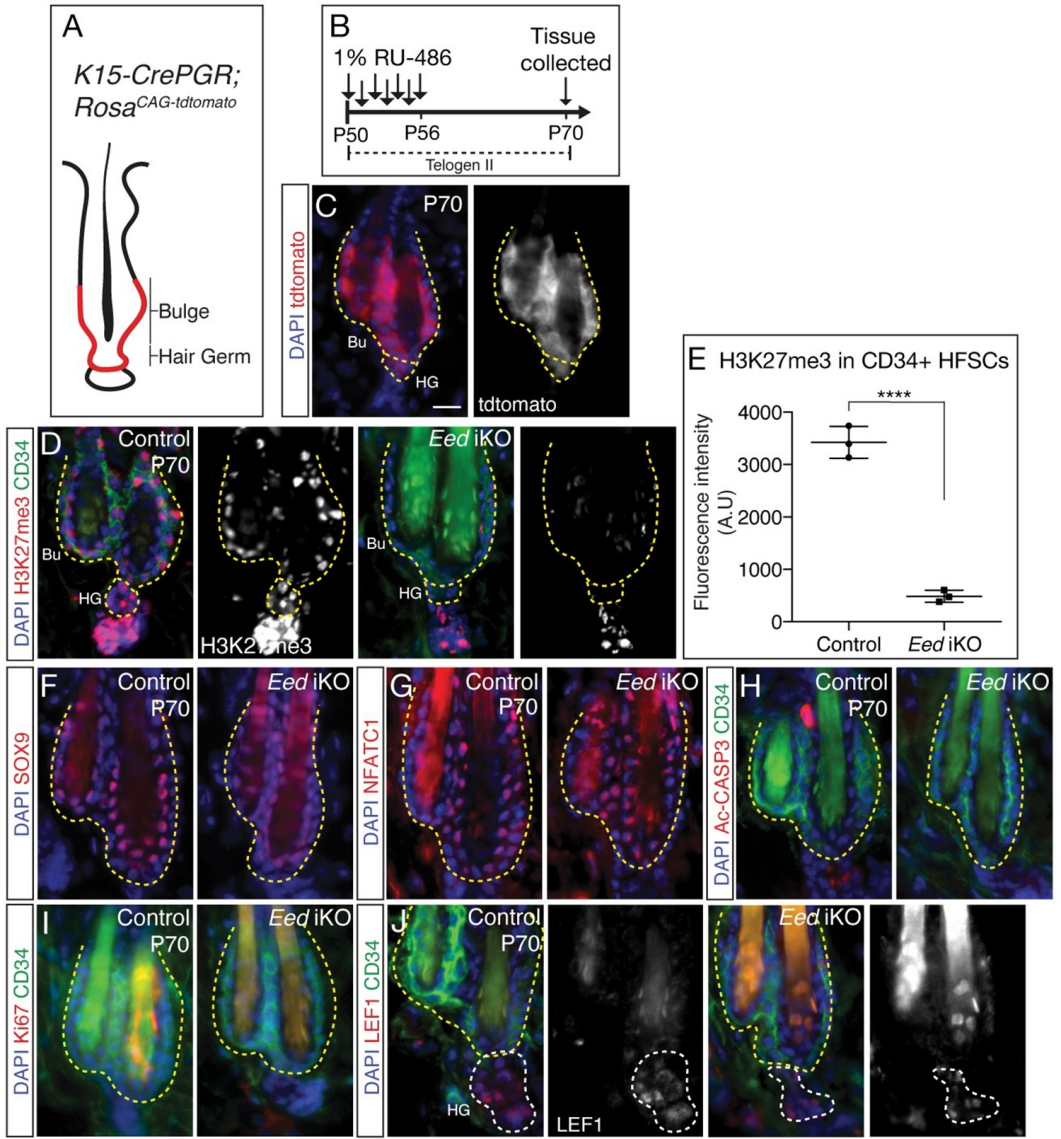
**HFSCs are maintained upon PRC2 loss (A)** Schematic illustrating expected tdtomato expression in the HF bulge of *K15-CrePGR; Rosa*^*CAG-tdtomato*^ mice. **(B)** Schematic showing the experimental strategy to induce *K15-CrePGR* activity in telogen II (P50) HFs. (**C**) IF analyses of tdtomato (red) and DAPI (blue) in *K15-CrePGR; Rosa*^*CAG-tdtomato*^ mice showing efficient targeting of the HF bulge. **(D)** IF analysis of H3K27me3 (red), CD34 (green) and DAPI (blue) in HFSCs of P70 control and *Eed* iKO mice. H3K27me3 single channel is shown in gray. Bulge (Bu) and hair germ (HG) regions of the follicle has been outlined in yellow. **(E)** Fluorescence intensity quantification of H3K27me3 signal (arbitrary units) in control and *Eed*-null HFSCs *P*<0.00001, *n* = 120 cells from 15 HF sections from three independent biological replicates for each group. **(F-J)** IF analyses of (F) SOX9 (red) and DAPI (blue), (G) NFATC1 (red) and DAPI (blue), (H) activated-CASPASE3 (red), CD34 (green) and DAPI (blue), (I) Ki67 (red), CD34 (green) and DAPI (blue) and (J) LEF1 (red), CD34 (green) and DAPI (blue) in HFs of P70 control and *Eed* iKO mice. LEF1 single channel is shown in gray. All IF experiments in this figure was conducted on skin sections collected from three animals for each group from two independent litters. Scale bar: 10μm.

To carry out functional studies investigating the role of PRC2 in the HF bulge, which consists of the CD34+ HFSCs and the differentiation committed stem cells of the hair germ (HG), we utilized the *K15-CrePGR* mouse line [15] (Fig 1A) and crossed it with *Eed*^*flox/flox*^ mice to generate *K15-CrePGR*: *Eed*^*fl/fl*^ (*Eed* iKO) mice. In these animals, topical application of RU-486 induces ablation of a key PRC2 core subunit, EED, which is sufficient to abrogate PRC2 function [16]. We confirmed efficient targeting of the HF bulge using the *K15-CrePGR* line by crossing it with (ROSA)^CAG-tdTomato^ reporter system. Topical application of RU-486 for seven days during telogen II when HFSCs stay in quiescent state for several weeks was efficient in driving Tdtomato expression in the HF bulge (Fig 1B and 1C). To ascertain that application of RU-486 was efficient in ablating H3K27me3, we carried out immunofluorescence (IF) analysis of H3K27me3, co-stained with a HFSC marker CD34. We observed significant erasure of H3K27me3 by P57 and further ablation by P70 in *Eed*-null HFSCs compared to controls (Figs 1D, 1E and S1A–S1C). Interestingly, prior work in intestinal stem cells (ISCs) had shown that effective loss of H3K27me3 occurs over several cellular divisions upon ablation of PRC2 function [17]. Comparatively, H3K27me3 is significantly erased in qHFSCs that do not undergo proliferation.

Next, we asked if loss of PRC2 and H3K27me3 ablation leads to alteration of HFSC identity and function. IF analysis of a HFSC marker SOX9, and a HFSC quiescent marker NFATC1 showed no noticeable changes between control and *Eed*-null HFSCs suggesting that HFSC identity is maintained upon *Eed* loss (Fig 1F and 1G). Next, we carried out IF analysis of a pro-apoptotic marker P19 (ARF) that is encoded by the *Cdkn2a* gene, a known target of PRC2 repression [18,19]. We observed that loss of PRC2 in the HFSCs does not lead to expression of P19 (S1D Fig) compared to *Eed*-null epidermis where P19 is expressed in the basal cells (S1E Fig) [20]. We also did not observe programmed cell death when assayed for activated Caspase 3 (Fig 1H).

Lastly, ChIP-seq studies of H3K27me3 has shown that many genes expressed in the differentiating and proliferating HF-TACs are demarcated by H3K27me3 in qHFSCs suggesting that loss of PRC2 and H3K27me3 in the qHFSCs would lead to expression of H3K27me3 demarcated HF-TAC genes which in turn would lead to premature loss and/or activation of HFSCs [14]. Therefore, to test whether loss of PRC2 in HFSCs results in premature activation we carried out IF analysis of a proliferative marker Ki67 and showed no premature activation of *Eed*-null HFSCs (Fig 1I). Next, we asked whether HF-TAC genes are activated in *Eed*-null HFSCs by carrying out IF analysis of a HF-TAC marker LEF1, a H3K27me3 target in the HFSCs [14]. We found that although LEF1 was expressed in HG cells, it was not expressed in the CD34+ *Eed*-null HFSCs similar to that of control HFSCs (Fig 1J). Together, these analyses show that loss of H3K27me3 in HFSCs does not alter HFSC quiescence or cell fate and does not lead to the induction of HF-TAC genes.

### Loss of H3K27me3 in HFSCs is not sufficient to induce HFSC activation or fate switch

**Fig 2:**
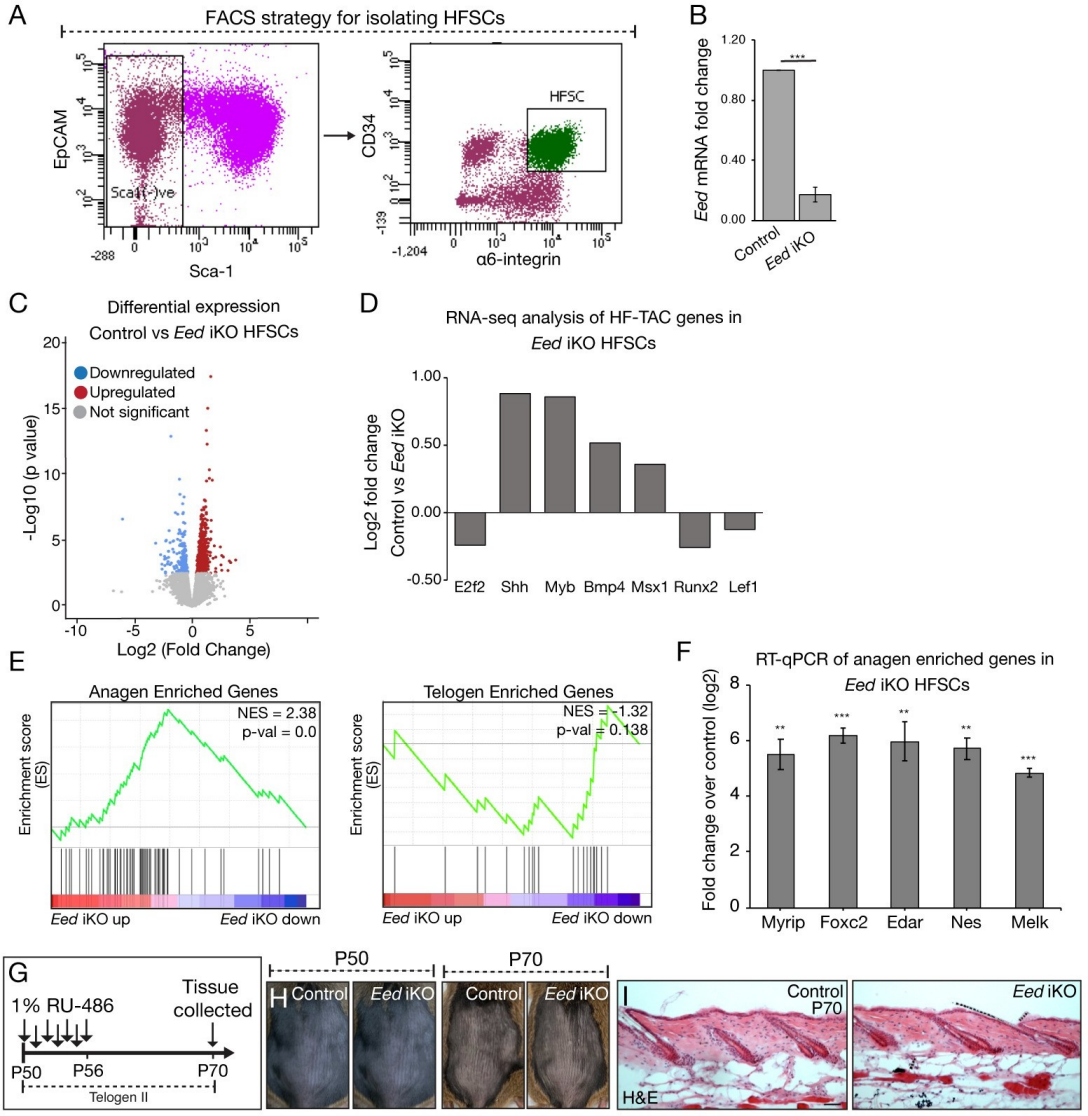
**Loss of PRC2 leads to expression of a subset of anagen enriched genes (A)** FACS strategy for isolating HFSCs from the back skin of mice. **(B)** RT-qPCR analysis of *Eed* mRNA in HFSCs isolated from control and *Eed* iKO mice. n = 3. ***p-value < 0.001; Data are mean ± SE. Three biological replicates for each group were used from at least two independent litters. **(C)** Volcano Plot depicting the differentially expressed genes in FACS-purified *Eed* iKO HFSCs vs corresponding control. Genes with absolute fold change ≥ 1.5 and adjusted p-value < 0.05 were considered significantly upregulated or downregulated for further analysis. RNA-seq analysis was done on three biological replicates for each group from at least two independent litters. **(D)** Graph depicting RNA-seq analysis of H3K27me3 demarcated HF-TACs genes in *Eed* iKO HFSCs. Fold change of these genes was not significant. **(E)** Gene set enrichment analysis of genes differentially expressed in *Eed* iKO HFSCs with anagen or telogen enriched genes. There was significant enrichment with anagen enriched gene set with p-value <0.05. Anagen and telogen enriched gene dataset was obtained by conducting RNA-seq on FACS purified HFSCs of six P28-30 and P50-52 animals, respectively. **(F)** RT-qPCR analysis of anagen enriched genes and H3K27me3 targets that are upregulated in HFSCs of *Eed* iKO mice. Three biological replicates for each group were used from at least two separate litters. ***p-value < 0.001, **p-value < 0.01; Data are mean ± SE. **(G)** Schematic showing the experimental strategy to ablate *Eed* in quiescent HFSCs in telogen II (P50) HFs. **(H)** Images of back skin of control and *Eed* iKO mice on P50 (day 1 of treatment) and P70 (day 20 post treatment). **(I)** Hematoxylin and Eosin (H&E) analysis of back skins from P70 control and *Eed* iKO mice. Analysis was done on three independent biological replicates for each group from two separate litters. Scale bar for H&E: 50μm.

To identify the transcriptional changes occurring in HFSCs lacking H3K27me3 we isolated HFSCs from P70 *Eed* iKO and control mice by fluorescence-activated cell sorting (FACS). HFSCs were isolated as EpCAM^+^, Sca1^-^, α6-integrin^high^, CD34^+^ cells as previously described (Fig 2A) [14]. qRT-PCR analysis confirmed downregulation of *Eed* mRNA in *Eed*-null isolated HFSCs compared to control (Fig 2B). We next subjected isolated RNAs to high-throughput sequencing (RNA-seq) and performed differential gene expression analysis that revealed significant upregulation of 213 genes and significant downregulation of 216 genes in *Eed*-null HFSCs when compared to control HFSCs (Fig 2C and S1 Table). Notably, out of the 213 significantly upregulated genes, only 54 were direct targets of H3K27me3 in qHFSCs (S2A Fig). Further analysis showed that HF-TAC genes that are demarcated by H3K27me3 in HFSCs [14] were not among the significantly upregulated genes in *Eed* iKO HFSCs (Fig 2D), concurring our IF data (Fig 1J) and bolstering the observation that loss of H3K27me3 in the HFSCs is not sufficient to induce transcription of these fate-switch genes. Moreover, out of the 468 bivalent genes demarcated by both H3K4me3 and H3K27me3 in qHFSCs [14], only 7 genes were upregulated in *Eed*-null HFSCs (S2B Fig). This suggests that loss of PRC2 function is not sufficient to activate bivalent genes in qHFSCs and other regulatory processes maintain their repression.

Although we did not observe activation of TAC genes in *Eed* iKO HFSCs, we asked whether loss of PRC2 in quiescent HFSCs leads to induction of genes expressed in aHFSCs. To answer this, we first performed RNA-seq analysis of FACS-purified HFSCs isolated from the back skin of P28-30 (anagen) and P50-52 (telogen) wild type mice. Differential gene expression analysis revealed 770 genes that are significantly upregulated in aHFSCs (anagen enriched genes) and 735 are significantly upregulated in qHFSCs (telogen enriched genes) (S2 Table). We then performed a Gene Set Enrichment Analysis (GSEA) and found that a subset of genes that are upregulated in *Eed* iKO HFSCs are anagen enriched genes (Fig 2E, left). On the contrary, there was no significant enrichment of telogen enriched genes in *Eed* iKO HFSCs (Fig 2E, right). Notably, out of the 47 anagen enriched genes that were upregulated in *Eed* iKO HFSCs, 19 are H3K27me3 targets in qHFSCs. We confirmed the upregulation of selected anagen enriched genes in the *Eed* iKO HFSCs via RT-qPCR (Fig 2F). We also carried out IF analysis of anagen enriched genes, EDAR and FOXC2, and found that both proteins were expressed in CD34+ HFSCs of *Eed* iKO but not control mice (S2C and S2D Fig). Lastly, to phenotypically observe whether PRC2 ablation in the HFSCs leads to shortened quiescence and premature onset of anagen, we induced PRC2 ablation during the telogen II (P50) when HFSCs stay in quiescent state for several weeks (Fig 2G). Similar to control animals, none of the *Eed* iKO animals exhibited hair growth 20 days post treatment and Hematoxylin and Eosin (H&E) analysis showed that HFs in both control and *Eed* iKO mice remained in telogen (Fig 2H and 2I). Together, these combined analyses show that loss of PRC2 in qHFSCs leads to an upregulation of subset of genes that are normally expressed in aHFSCs. However, expression of these genes in *Eed*-null HFSCs is not sufficient to override the quiescent transcriptional program which would manifest into phenotypic changes.

### HFSCs lacking PRC2 undergo proper hair regeneration

**Fig 3:**
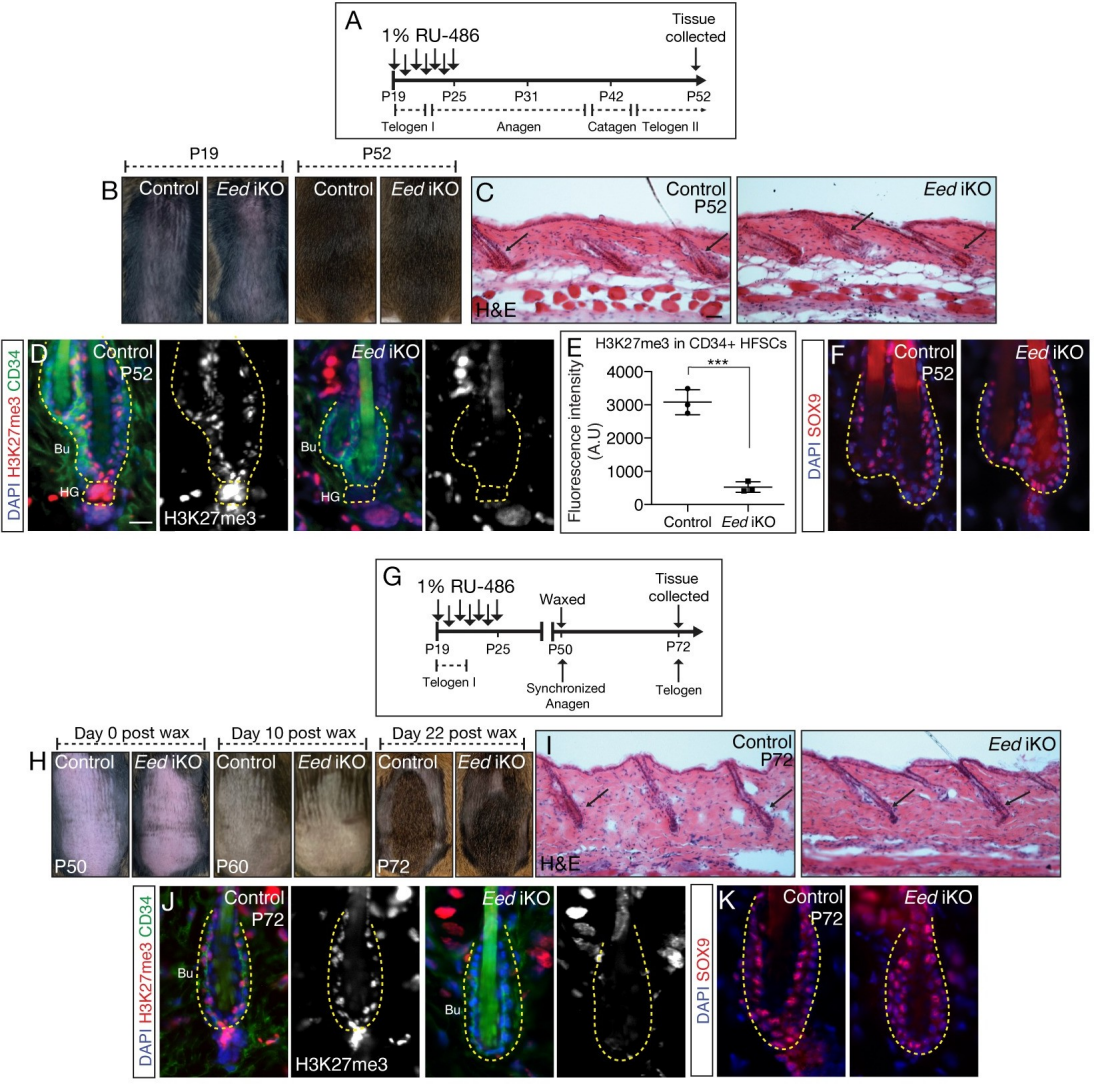
**HFSCs lacking PRC2 progress normally through hair cycle and form new HF bulge (A)** Schematic showing the experimental strategy to ablate *Eed* in quiescent HFSCs in telogen I HFs. **(B)** Images of back skin of control and *Eed* iKO mice on P19 (day 1 of treatment) and P52 (day 27 post treatment). **(C)** H&E analysis of P52 control and *Eed* iKO back skin. Club hairs are indicated by arrows. **(D)** IF analysis of H3K27me3 (red), CD34 (green) and DAPI (blue) in HFSCs of P52 control and *Eed* iKO mice. H3K27me3 single channel is shown in gray. **(E)** Fluorescence intensity quantification of H3K27me3 signal (arbitrary units) in control and *Eed*-null HFSCs *P*<0.0001, *n* = 100 cells from 10–12 HFs from at least three independent biological replicates. **(F)** IF analyses of SOX9 (red) and DAPI (blue) in HFSCs of P52 control and *Eed* iKO mice. **(G)** Schematic showing the experimental strategy to ablate *Eed* in telogen I (P19) HFSCs followed by waxing (depilation) at P50 to induce regeneration. **(H)** Images of waxed back skin of control and *Eed* iKO mice on day 0, day 10, and day 22 after waxing. **(I)** H&E analysis of skins collected 22 days post waxing from control and *Eed* iKO back skin. **(J)** IF analysis of H3K27me3 (red), CD34 (green) and DAPI (blue) in HFSCs of 22 days post waxed skin from control and *Eed* iKO mice. H3K27me3 single channel is shown in gray. **(K)** IF analyses of SOX9 (red) and DAPI (blue) in HFSCs of P72 control and *Eed* iKO mice. All reported experiments in this figure was conducted on three biological replicates from two separate litters. Scale bar for H&E: 50μm, Scale bar for IF: 10μm.

Given that loss of PRC2 function in qHFSC does not instructively affect HFSC function, we asked whether ablation of PRC2 in the HFSCs would impact hair cycle and hair regeneration. Therefore, we ablated *Eed* at P19 during the quiescent stage of the first adult hair cycle, i.e., telogen I (Fig 3A). To confirm that H3K27me3 was ablated in HFSCs at the end of this treatment regimen, we collected dorsal skin from animals at P26 and confirmed that H3K27me3 mark was significantly ablated in *Eed*-null HFSCs when compared to control (S3A–S3C Fig). Next, we followed homeostatic hair cycle in control and *Eed* iKO animals after treatment between P19-P25 and found that both the animals had undergone proper hair regeneration and exhibited a full hair coat (Fig 3B). Next, we collected the back skin at P52 to test whether there were any histological differences in the HFs between control and *Eed* iKO mice. H&E staining showed no gross differences in HF morphology in control and *Eed* iKO mice. Moreover, both animals had completed a full homeostatic hair cycle to enter telogen II indicated by the presence of club hairs with old and newly formed bulge (Fig 3C). IF analysis of P52 *Eed* iKO HFs showed that the new bulge was devoid of H3K27me3 mark when compared to control (Fig 3D and 3E). Additionally, IF analysis of SOX9 showed no noticeable changes between control and *Eed*-null HFSCs suggesting that HFSC identity was properly re-instated in the newly formed HFSCs of the new bulge (Fig 3F). Next, we let these animals undergo two homeostatic hair cycles and observed no defects in hair regeneration or changes in HFSC fate in *Eed* iKO mice when compared to control (S3D–S3H Fig). Lastly, we challenged PRC2-null HFSCs to undergo forced regeneration brought upon by depilation (waxing) [21]. Once control and *Eed*-null HFs had entered the quiescent period at P50, we waxed the back skin to induce hair cycle (Fig 3G). We found no changes in rate of hair regeneration between control and *Eed* iKO mice and H&E staining of skin 22 days post waxing showed that both control and *Eed* iKO mice had not only undergone induced anagen but had successfully completed hair cycle to re-enter telogen (Fig 3H and 3I). IF analysis confirmed that HFSCs remained devoid of H3K27me3 in *Eed* iKO mice (Fig 3J). Additionally, there were no changes in SOX9 expression between control and *Eed*-null HFSCs (Fig 3K). Altogether, these observations led us to conclude PRC2 function is dispensable for hair regeneration.

## Discussion

Histone modifications provide a critical cell-intrinsic mechanism by which stem cells regulate its transcriptional landscape to either maintain its SC identity or initiate a differentiation program [22,23]. However, *in vivo* functional studies testing the role of specific chromatin regulators in SC systems of adult tissue are limited. In this report we uncover that a conserved epigenetic regulator, PRC2 and its corresponding repressive histone mark H3K27me3, is dispensable for HFSC quiescence and HF regeneration. We show that *in vivo* genetic ablation of PRC2 in the HFSCs does not impair HFSC function and hair cycle. Further analysis of quiescent HFSCs revealed that loss of PRC2 does not lead to expression of genes expressed in differentiating HF-TACs that have been reported to be H3K27me3 bound in the HFSCs [14]. Lastly, transcriptional profiling of quiescent HFSCs lacking PRC2 revealed loss of H3K27me3-mediated repression leads to expression of genes that are typically expressed in aHFSCs. Together, our findings show that ablation of PRC2 in the adult HFSCs leads to modest transcriptional changes that are associated with the activated state of the HFSCs and that is insufficient to alter HFSC fate or function to have physiologically relevant effects. Notably, our finding is in line with previously reported observation that PRC2 function is largely dispensable for maintaining intestinal stem cells (ISCs) and homeostatic intestinal regeneration in adult mice, further elucidating the non-instructive role of PRC2 in SCs of different epithelial tissue of an adult organism [24].

We had previously reported that PRC2 is required for postnatal development of HFs [10,11]. This defect was due to defective proliferation and increased apoptosis in highly proliferative HF matrix cells leading to arrested HF formation. Importantly, despite the defective formation of HFs, HFSC specification was not altered [10,11]. Here, we report that ablation of PRC2 function in the adult HFSCs does not impact hair regeneration or HFSC state. Therefore, from our studies in development and now in adulthood, we can conclude that PRC2 and H3K27me3 is dispensable for HFSC specification during development and HFSC function during hair regeneration in the adult skin. Interestingly, loss of function of JARID2, a key PRC2 complex member, during epidermal development does not affect basal cell differentiation and function, nor does it impact HF morphogenesis [25]. On other hand, loss of *Jarid2* in the adult epidermis leads to reduced proliferation and enhanced differentiation of basal cells leading to abnormal epidermal thickness. Moreover, there is no impact on hair cycle progression [25]. This indicates that PRC2 complex containing JARID2 has a more instructive role in the interfollicular epithelium and is dispensable during development. Additionally, our lab has recently reported that loss of PRC2 function in adult epidermal stem cells leads to gross phenotypic changes including epidermal pigmentation which is not observed when PRC2 function is ablated exclusively in the HF bulge [20]. Together, these studies highlight that the instructive function of PRC2 not only varies between different cell populations of the developing and adult epidermis, but its function is context dependent.

Cell fate specification controlled by epigenetic regulatory mechanisms can span many cellular divisions–especially histone methylation has been considered to enforce its mode of gene regulation over multiple rounds of cell division [26]. Studies in ISCs reported that ablation of PRC2 does not lead to immediate loss of H3K27me3 and de-repression of PRC2 targets. Rather H3K27me3 dilutes on parental nucleosomes over several rounds of division leading to full activation of genes under PRC2 repression [17]. This is contrary to what we observed in HFSCs as complete loss of H3K27me3 in HFSCs occurred as cells remained quiescent (Fig 2D). Notably, prior work has shown that Jmjd3, a Jumonji family of de-methylases, which removes methyl groups from H3K27 to alleviate Polycomb mediated repression, is expressed in the HFSCs [13,27]. Together, these data indicate that complete erasure of H3K27me3 in HFSCs is mediated by not only the loss of the methyltransferase activity of PRC2 but also by swift and uninhibited activity of de-methylases. Therefore, while the mild phenotypes seen upon PRC2 loss in ISCs can be attributed to gradual de-repression of target genes through cell division dependent H3K27me3 dilution, insufficient erasure of H3K27me3 is not a contributing factor for the non-instructive role of PRC2 in HFSCs [24]. Interestingly, pharmacological inhibition of de-methylases in the epidermis, including the HFSCs, leads to defective hair regeneration and wound repair, suggesting that elevated levels of H3K27me3 mark, but not ablation, plays a more prominent role in the HFSCs [13,28].

The canonical model of gene regulation by Polycomb proteins postulates that PRC1 and PRC2 facilitate each other’s recruitment and largely bind to the same targets to establish Polycomb domains to mediate transcriptional repression [8,29]. However, very little was known of how each complex contributes to gene repression and if their function is redundant or not *in vivo*. Our recent work had highlighted that PRC1 and PRC2 have functional redundancy in epidermal stem cells during development, and this redundant repressive role is imperative for preserving epidermal lineage identity [12]. Given that loss of PRC2 alone in the HFSCs does not have an instructive role, we speculate that in absence of PRC2, PRC1 might be compensating to maintain stem cell identity and quiescence. Notably, we observed that the *Cdkn2a* locus, a target of Polycomb repression, is not de-regulated in the *Eed*-null HFSCs also implying that PRC1 maybe maintaining repression in absence of PRC2 [18,19]. Therefore, it will be intriguing to investigate whether PRC2 targets in the HFSCs are also targeted by PRC1, and whether loss of both PRC1 and PRC2 can alleviate this repression to reprogram the HFSCs to acquire a cell fate that is more committed to differentiation. Furthermore, there are other chromatin regulatory mechanisms that have been implicated in HFSCs and that could be safeguarding HFSC function in absence of PRC2 function [4,30]. Ablation of histone deacetylase 1/2 (HDAC1/2) function in the developing and adult epidermis leads to several HF defects such as absence of HF formation, expansion of HF bulge region, and alopecia [31–33]. Loss of DNA-methyltransferase 1 (DNMT1) in the developing epidermis results in improper HF architecture and progressive alopecia in aged animals [34]. Additionally, higher order chromatin organization and have also been linked to HFSC function. Super-enhancers (SEs) are clusters of closely associated enhancers that are bound by transcription factors involved in SC identity and fate choice [35]. In HFSCs, SEs are specifically associated with HFSC factors and undergo dynamic remodeling during HFSC lineage progression allowing for a specific transcriptional landscape that promotes fate switch processes [36].

Deregulation of epigenetic mechanisms often lead to the development of cancers. Loss-of-function mutations affecting PRC2 function has been associated with a myriad of cancers highlighting the tumor-suppressive roles of PRC2 [37]. Previous work has identified that HFSCs are the cells of origin of squamous cell carcinoma (SCC) and over-expression of oncogenic factors in the HFSCs is sufficient to induce SCC tumorigenesis [38,39]. Given that loss of PRC2 in HFSCs leads to an activated state of the cells, it could be speculated that loss of PRC2 could prime the HFSCs for malignant transformation contributing towards SCC formation. Although loss of PRC2 in the HFSCs does not lead to phenotypic changes that grossly affect hair regeneration, it will be interesting to see if application of chemical carcinogenic compounds would accelerate the initiation of SCC tumorigenesis in HFSCs lacking PRC2 function.

## Methods and materials

### Ethics statement

All experimental protocols using animals was approved by and in accordance with the Institutional Animal Care and Use Committee (IACUC) guidelines (Protocol No. LA11-00020).

### Mice

All mice used in this study were housed at the Center of Comparative Medicine and Surgery (CCMS), Icahn School of Medicine at Mount Sinai (ISMMS). *K15-CrePGR* and *Rosa*^*CAG-tdtomato*^ mice lines were obtained from the Jackson Laboratories. Drs. Weipeng Mu and Terry Magnuson [16] kindly provided the *Eed*^*flox/flox*^ mice. Mice were genotyped by PCR using tail DNA that was extracted using DirectPCR Lysis Reagent (Viagen Biotech Inc), according to manufacturer’s instructions. Both male and female mice were used in this study. For RU-486 (Cayman Chemicals) topical treatment to induce *Eed* ablation, RU-486 was dissolved in 100% ethanol (Sigma-Aldrich) to a final concentration of 10 mg/ml. 100μl of RU-486 was topically applied on shaved dorsal skin of postnatal day 19 (P19) or P50 mice once per day for 7 days. The back skin of P50 mice were waxed using Sally Hansen facial wax strips. Control mice were treated with the same amount of 100% ethanol.

### Immunofluorescence staining and microscopy

Dorsal skin tissues were collected from adult mice, embedded in OCT compound (Tissue-Tek), and subsequently cut into 8μm sections using a Leica Cryostat. Slides with sections were fixed in 4% PFA for 15 minutes at room temperature and blocked for 1 hour at room temperature in blocking solution (1x PBS supplemented with 0.1% Triton X-100, 1% BSA, 0.25% normal donkey serum, 0.01% gelatin). Primary antibodies were diluted in blocking solution and incubated for overnight at 4°C. Slides were then incubated with secondary antibodies for 1 hour at room temperature. Slides were counterstained with DAPI to visualize nuclei. The following primary antibodies were used in this study: Rabbit anti-H3K27me3 (1:500, Cell Signaling, Cat# 9733S), Rat anti-CD34 (1:100, eBioscience, Cat# 14-0341-82), Rabbit anti-KI67 (1:250, Abcam, Cat# ab15580), Rabbit anti-activated caspase 3 (1:250, R&D Systems, Cat# AF835), Rabbit anti-SOX9 (1:250, Abcam, Cat# ab185966), Mouse anti-NFATC1 (1:100, Biolegend, Cat#649601), Rabbit anti-LEF1 (1:100, Cell Signaling, Cat# 2230S), Rabbit anti-P19 (ARF) (1:200, Abcam, Cat#ab80), Goat anti-EDAR (1:100, R&D Systems, Cat# AF745) and Sheep anti-FOXC2 (1:100, R&D Systems, Cat# AF6989). The following Secondary antibodies from Jackson ImmunoResearch Labs were used in this study: Anti-rat Alexa Fluor 488 (Cat# 712-545-150), Anti-mouse Alexa Fluor 594 (Cat# 711-585-152), Anti-rabbit Alexa Fluor 594 (Cat# 711-585-152), Anti-rabbit Alexa Fluor 647 (Cat# 711-605-152) and Anti-goat Alexa Fluor 647 (Cat# 705-606-147). Anti-sheep NL557 (Cat# NL010) was acquired from R&D Systems.

Slides were imaged using a Leica DM5500 slide microscope. For each immunofluorescence assay, at least three animals from at least two independent litters were analyzed per mutant genotype.

### Immunofluorescence quantification and statistics

Slides were imaged using Leica DM5500 slide microscope with 40X objective. Fluorescence intensity for each time point was calculated from at least 15 bulge images totaling >100 HFSCs.

NIH ImageJ software was used to calculate the mean intensity for H3K27me3. Fluorescence intensity was normalized to non-nuclear background. Graphs were generated using GraphPad Prism software. Each point on the column graph indicates average of each biological replicate. The error bars represent st. dev. To determine the significance between two groups, comparisons were made using Student’s *t*-test (Excel). For all statistical tests, the 0.05 level of confidence was accepted for statistical significance. **, *** and **** indicates p-values < 0.001, <0.0001 and < 0.00001, respectively.

### Fluorescence-activated cell sorting

FACS isolation of HFSCs from the back skin of control and *Eed* iKO mice was performed as following. The back skin from adult mice was collected. The adipose layer from the dorsal side was scraped off and the scraped skin sample was washed with 1x PBS prior to incubation with 0.25% Trypsin/EDTA (Corning Cellgro) at 37°C for 1 hour. After incubation, the epidermal cells, including the HFSCs, was scraped off from the trypsinized skin into the plate. 25 mLs of E-media was added to the cell suspension and was strained through 40μm filters and was washed twice with 1x DPBS. The cells were stained with 1:200 PerCP-Cy5.5-Sca1 (Thermo Fisher Scientific), 1:200 FITC-α6-integrin (Thermo Fisher Scientific), 1:100 APC-EpCAM (Biolegend) and 1:20 Alexa700-CD34 (Biolegend) in staining buffer (HBSS + 2% Fetal Bovine Serum) for 30 minutes on ice and then washed twice with 1x DPBS before cell sorting. HFSCs were sorted by gating on EpCAM(+), Sca1(-), α6-integrin(high) and CD34(+). All cell isolations were performed on a FACS Influx instrument (BD) in the Flow Cytometry Core Facility at ISMMS.

### RNA purification, RT-qPCR, and RNA-seq library preparation

FACS-purified cells were collected directly into RLT Plus buffer (QIAGEN) and total RNA was isolated with the RNeasy Plus Micro Kit (QIAGEN) and DNase1 treatment (QIAGEN). cDNA was reverse transcribed from total RNA using qScript cDNA SuperMix (Quanta Biosciences) according to the manufacturer’s instructions. Samples were analyzed by RT-qPCR using LightCycler 480 SYBR Green I Master Mix (Roche) on a Lightcycler 480 instrument (Roche). For RNA-seq library preparation, 10ng of total RNA was reverse transcribed and amplified using the Ovation RNA-seq System V2 (NuGEN). Libraries were constructed from 200ng of sonicated cDNA (Covaris M220, Covairs) using the Ovation Ultralow system V2 (NuGEN). The concentration and quality of the libraries were determined using Qubit (Invitrogen) and Bioanalyzer (Agilent). Constructed RNA-seq libraries were sequenced at GENEWIZ on the Illumina HiSeq platform, obtaining 150 nucleotide paired-end reads.

### RT-qPCR analysis and statistics

To calculate fold change of mRNA levels of anagen enriched genes (AEGs) in *Eed*-null HFSCs, first, the Ct values of technical replicates of each trial was averaged. DCt was calculated by subtracting GAPDH Ct average from the Ct average of AEGs. Then the of the 2^-DCt was calculated for each trial. Fold change was then calculated by dividing *Eed* iKO 2^-DCt by control 2^-DCt. Bar graph is presented as mean ± SD. At least three animals for each group from two independent litters were used. For all statistical tests, the 0.05 level of confidence was accepted for statistical significance. ** and *** indicates p-values < 0.001 and <0.0001, respectively. Primer details are available upon request to the corresponding author.

### RNA-seq data analysis

The RNA-seq reads were aligned to the mouse transcriptomes corresponding to the Gencode annotation (vM20) using the software Kallisto (v0.42.5). To determine differentially expressed genes with the software DESeq2 between control and *Eed* iKO protein-coding genes with an average TPM (transcripts per million reads mapped) > 1 in control and *Eed* iKO were used. Genes considered upregulated in *Eed*-null HFSCs had a log2 fold change >1.5 at significance level of p-value < 0.05. Similar analysis method was used to determine differentially expressed genes between telogen and anagen HFSCs. Genes with log2 fold change <2.0 at significance level of p-value < 0.05 were considered anagen enriched genes and genes with log2 fold change >2.0 at significance level of p-value < 0.05 were considered telogen enriched genes.

### Gene Set Enrichment Analysis (GSEA)

For GSEA analysis of *Eed* iKO HFSCs, first a list of differentially expressed genes was generated. Genes that had >/<1.5 log2 fold-change in expression with p-value<0.05 was ranked and used as input for the GSEA tool (GSEA_4.1.0). The gene sets for GSEA were curated by obtaining the differentially expressed gene list by performing RNA-seq on quiescent HFSCs (qHFSCs, P50-52) and activated HFSCs (aHFSCs, P28-30). Genes that were upregulated in aHFSCs and upregulated in qHFSCs were defined as Anagen enriched and Telogen enriched genes, respectively.

## Supporting information

S1 Fig **PRC2-null HFSCs do not undergo apoptosis (A)** Schematic showing the experimental strategy to induce *K15-CrePGR* activity in telogen II (P50) HFs. (**B**) IF analyses of H3K27me3 (red), CD34 (green) and DAPI (blue) in HFSCs of P57 control and *Eed* iKO mice. H3K27me3 single channel is shown in gray. Bulge (Bu) region has been outlined in yellow. **(C)** Fluorescence intensity quantification of H3K27me3 signal (arbitrary units) in control and *Eed*-null HFSCs *P*<0.001, *n* = 90 cells from 11–12 HF sections from two independent biological replicates for each group. **(D)** IF analyses of P19^Arf^ (red), CD34 (green) and DAPI (blue) in HFSCs of P70 control and *Eed* iKO mice. Bulge (Bu) region has been outlined in yellow **(E)** IF analyses of P19^Arf^ (red) and DAPI (blue) in the epidermis of P70 *K14-CreERT2; Eed* mice. Epidermis has been separated from dermis region with yellow dashed line. All P70 IF analysis was conducted on three biological replicates for each group from two separate litters. Scale bar for IF: 10μm.
(TIF)

S2 Fig **Loss of PRC2 leads to expression of a subset of anagen enriched genes (A)** Venn diagram showing number of shared genes that are H3K27me3 demarcated in qHFSCs and are also upregulated in *Eed* iKO HFSCs. **(B)** Venn diagram showing number of shared genes that are bivalent in qHFSCs and upregulated in *Eed* iKO HFSCs. **(C)** IF analyses of EDAR (red), CD34 (green) and DAPI (blue) in HFSCs of P70 control and *Eed* iKO mice. **(D)** IF analyses of FOXC2 (red), CD34 (green) and DAPI (blue) in HFSCs of P70 control and *Eed* iKO mice. Bulge (Bu) and hair germ (HG) region has been outlined in yellow. All P70 IF analysis was conducted on three biological replicates for each group from two separate litters. Scale bar for IF: 10μm.
(TIF)

S3 Fig **HFSCs lacking PRC2 undergo homeostatic hair regeneration (A)** Schematic showing the experimental strategy to induce *K15-CrePGR* activity in telogen I (P19) HFs. (**B**) IF analyses of H3K27me3 (red), CD34 (green) and DAPI (blue) in HFSCs of P26 control and *Eed* iKO mice. H3K27me3 single channel is shown in gray. Bulge (Bu) region has been outlined in yellow. **(C)** Fluorescence intensity quantification of H3K27me3 signal (arbitrary units) in control and *Eed*-null HFSCs *P*<0.0001, *n* = 110 cells from 12–13 HF sections from three independent biological replicates for each group. **(D)** Schematic showing the experimental strategy to induce *K15-CrePGR* activity in telogen I (P19) HFs and following the animals through two homeostatic hair cycles. **(E)** Images of back skin of control and *Eed* iKO mice at P19 (after shaving), P50, P50 (after shaving) and P110. **(F)** H&E analysis of skins from P110 control and *Eed* iKO back skin. **(G)** IF analysis of H3K27me3 (red), CD34 (green) and DAPI (blue) in HFSCs of P110 control and *Eed* iKO mice. H3K27me3 single channel is shown in gray. **(H)** IF analysis of SOX9 (red) and DAPI (blue) in HFSCs of P110 control and *Eed* iKO mice. Analysis was conducted on three biological replicates for each group from at least two separate litters. Scale bar for H&E: 50μm, Scale bar for IF: 10μm.
(TIF)

S1 Table Differentially expressed genes in Eed iKO vs Control HFSCs. Genes that were upregulated and downregulated with a log2 fold change >1.5 and <1.5 with significance of p<0.05 are included.
(XLSX)

S2 Table Differentially expressed genes in Telogen HFSCs vs Anagen HFSCs. Genes that were upregulated and downregulated with a log2 fold change >2.0 and <2.0 with significance of p<0.05 are included.
(XLSX)
