# Supplementary figures and images for: Polycomb repressive complex 2 in adult hair follicle stem cells is dispensable for hair regeneration
Source: PLoS Genet. 2021 Dec 14;17(12):e1009948. doi: 10.1371/journal.pgen.1009948 (PMC8670713; doi:10.1371/journal.pgen.1009948)

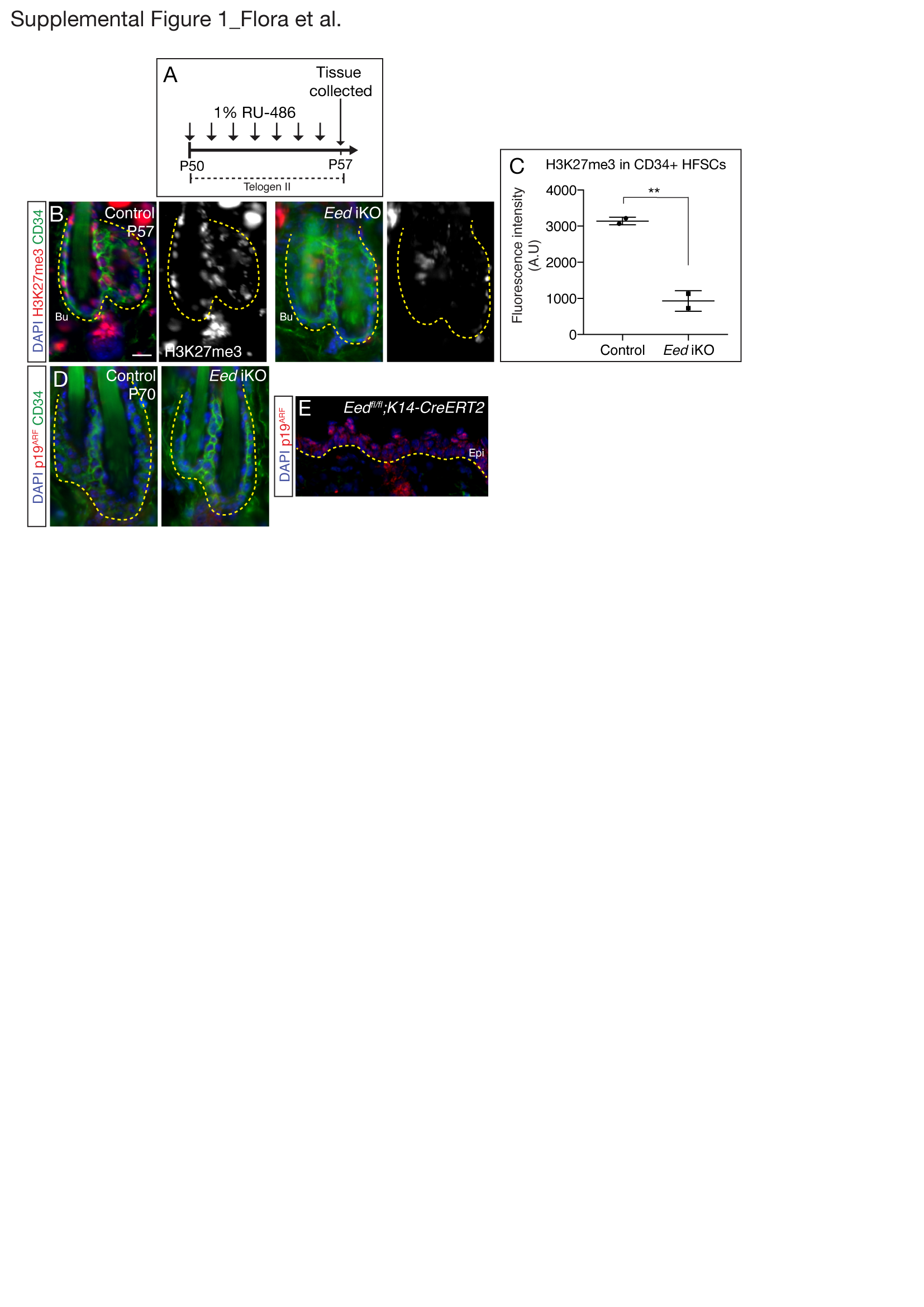

Supplement: S1 Fig — PRC2-null HFSCs do not undergo apoptosis (A) Schematic showing the experimental strategy to induce K15-CrePGR activity in telogen II (P50) HFs. (B) IF analyses of H3K27me3 (red), CD34 (green) and DAPI (blue) in HFSCs of P57 control and Eed iKO mice. H3K27me3 single channel is shown in gray. Bulge (Bu) region has been outlined in yellow. (C) Fluorescence intensity quantification of H3K27me3 signal (arbitrary units) in control and Eed-null HFSCs P<0.001, n = 90 cells from 11–12 HF sections from two independent biological replicates for each group. (D) IF analyses of P19Arf (red), CD34 (green) and DAPI (blue) in HFSCs of P70 control and Eed iKO mice. Bulge (Bu) region has been outlined in yellow (E) IF analyses of P19Arf (red) and DAPI (blue) in the epidermis of P70 K14-CreERT2; Eed mice. Epidermis has been separated from dermis region with yellow dashed line. All P70 IF analysis was conducted on three biological replicates for each group from two separate litters. Scale bar for IF: 10μm. (TIF) [file pgen.1009948.s001.tif]

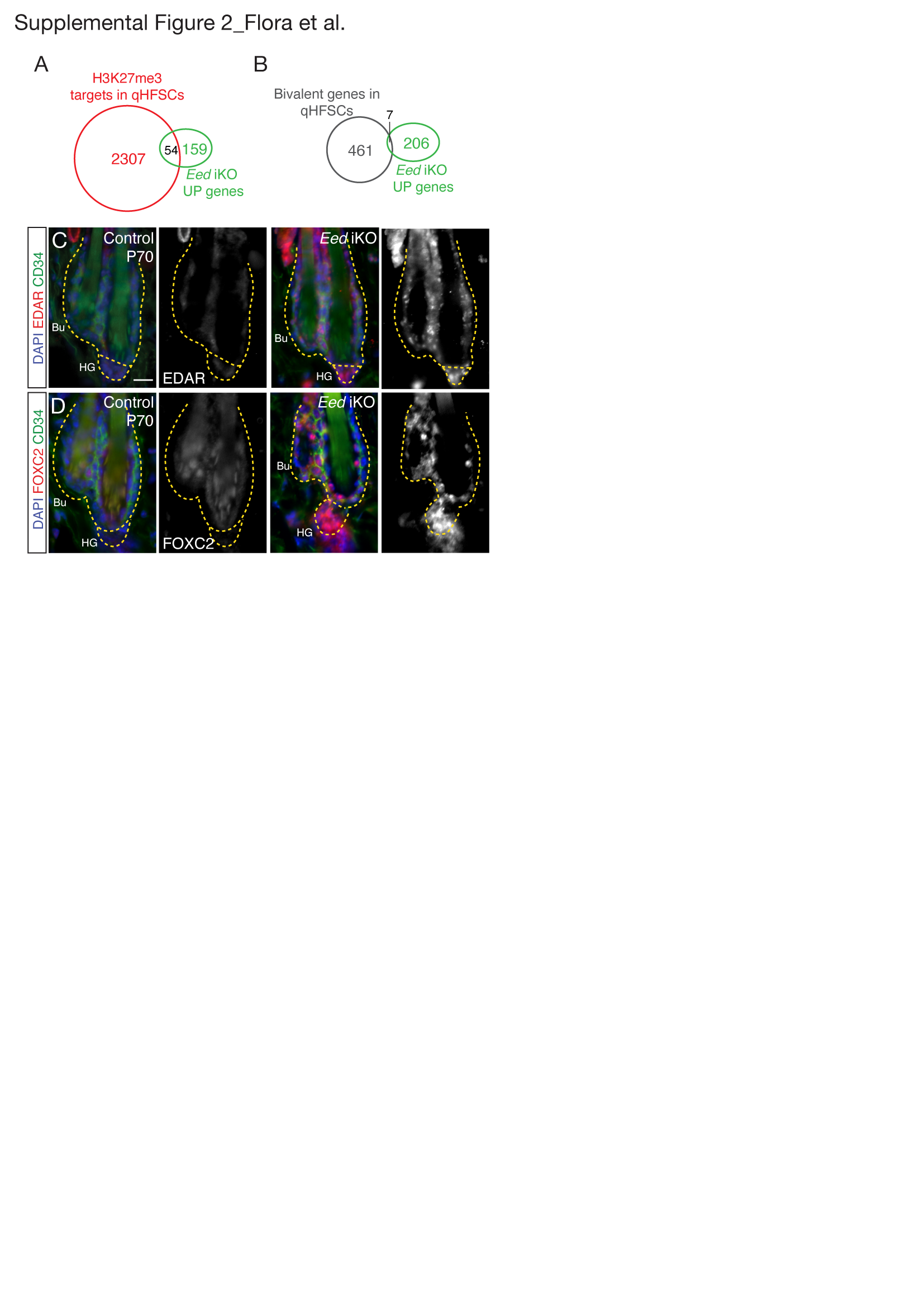

Supplement: S2 Fig — Loss of PRC2 leads to expression of a subset of anagen enriched genes (A) Venn diagram showing number of shared genes that are H3K27me3 demarcated in qHFSCs and are also upregulated in Eed iKO HFSCs. (B) Venn diagram showing number of shared genes that are bivalent in qHFSCs and upregulated in Eed iKO HFSCs. (C) IF analyses of EDAR (red), CD34 (green) and DAPI (blue) in HFSCs of P70 control and Eed iKO mice. (D) IF analyses of FOXC2 (red), CD34 (green) and DAPI (blue) in HFSCs of P70 control and Eed iKO mice. Bulge (Bu) and hair germ (HG) region has been outlined in yellow. All P70 IF analysis was conducted on three biological replicates for each group from two separate litters. Scale bar for IF: 10μm. (TIF) [file pgen.1009948.s002.tif]

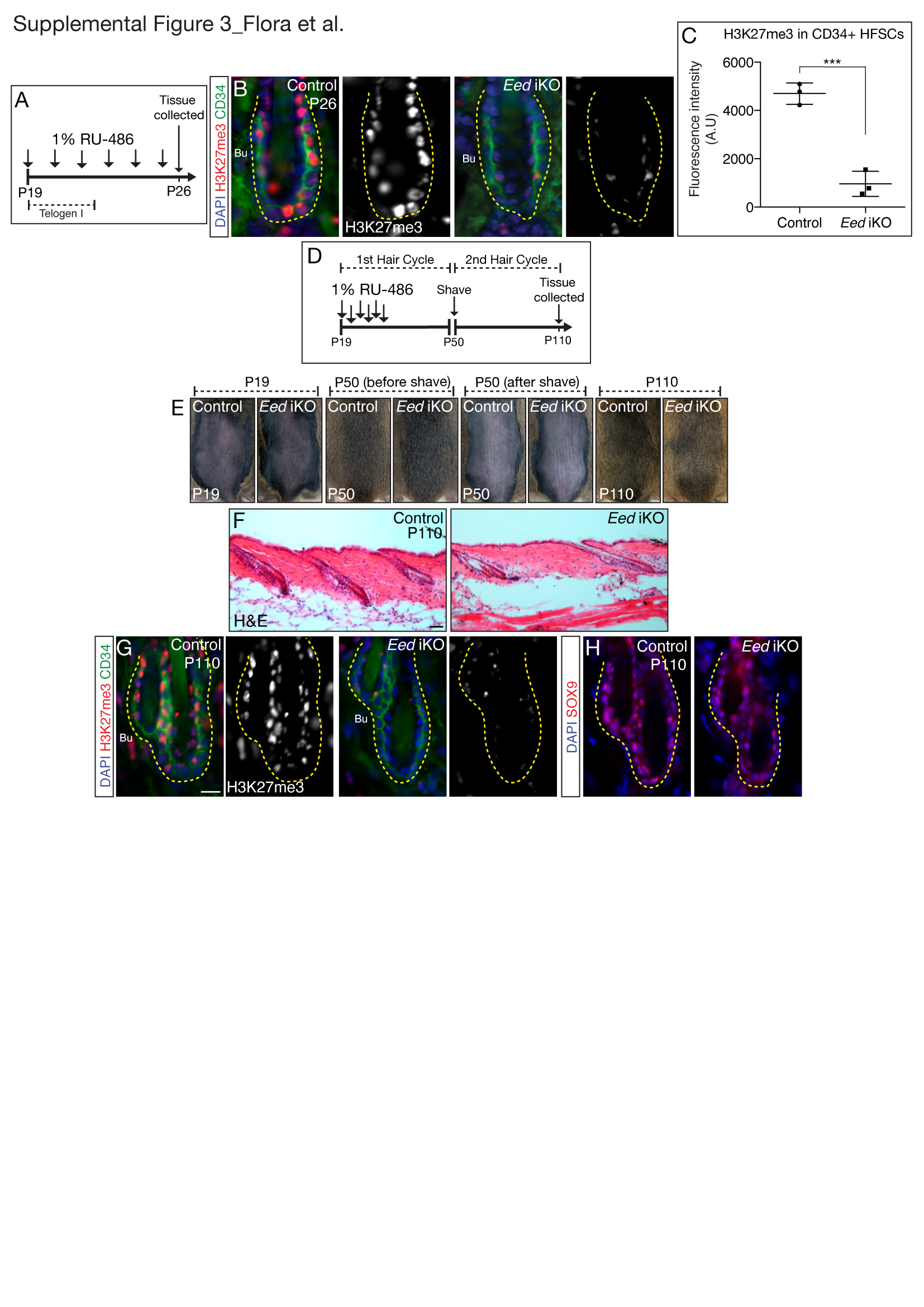

Supplement: S3 Fig — HFSCs lacking PRC2 undergo homeostatic hair regeneration (A) Schematic showing the experimental strategy to induce K15-CrePGR activity in telogen I (P19) HFs. (B) IF analyses of H3K27me3 (red), CD34 (green) and DAPI (blue) in HFSCs of P26 control and Eed iKO mice. H3K27me3 single channel is shown in gray. Bulge (Bu) region has been outlined in yellow. (C) Fluorescence intensity quantification of H3K27me3 signal (arbitrary units) in control and Eed-null HFSCs P<0.0001, n = 110 cells from 12–13 HF sections from three independent biological replicates for each group. (D) Schematic showing the experimental strategy to induce K15-CrePGR activity in telogen I (P19) HFs and following the animals through two homeostatic hair cycles. (E) Images of back skin of control and Eed iKO mice at P19 (after shaving), P50, P50 (after shaving) and P110. (F) H&E analysis of skins from P110 control and Eed iKO back skin. (G) IF analysis of H3K27me3 (red), CD34 (green) and DAPI (blue) in HFSCs of P110 control and Eed iKO mice. H3K27me3 single channel is shown in gray. (H) IF analysis of SOX9 (red) and DAPI (blue) in HFSCs of P110 control and Eed iKO mice. Analysis was conducted on three biological replicates for each group from at least two separate litters. Scale bar for H&E: 50μm, Scale bar for IF: 10μm. (TIF) [file pgen.1009948.s003.tif]
